# *Klebsiella oxytoca* P620 and *Pseudomonas* sp. CFA co-inoculation modulates rhizospheric bacterial communities to alleviate the combined stresses of phenolic acids and nitrate in cucumber

**DOI:** 10.3389/fmicb.2026.1834918

**Published:** 2026-06-10

**Authors:** Yan-Qing Zhu, Hongrui Bai, Pi-Yao Sun, Jiashuai Zhang, Jia-Yi Xu, Xin-Ying Zhang, Zhaoqian Liu, Caixia Xia, Wenhao Zhang, Xuan Liu, Jinguang Gao, Jiahe Zhao, Yi Zhang, Ji-Gang Bai, Xiu-Juan Wang

**Affiliations:** 1 College of Life Sciences, Shandong Agricultural University, Tai’an, Shandong, China; 2 Faculty of Pharmacy and Pharmaceutical Sciences, Monash University, Parkville, VIC, Australia

**Keywords:** bacterial community, co-inoculation, combined stress, cucumber, *Klebsiella*, nitrate, phenolic acid, *Pseudomonas*

## Abstract

Crops in greenhouses are subjected to the combined stresses of phenolic acids and nitrate; however, approaches to simultaneously mitigate these stresses have not been explored. In this study, p-hydroxybenzoic acid (PHBA)-degrading *Klebsiella oxytoca* P620 reduced nitrate to ammonium via nitrite through *narGHI* and *nirBD* genes. When the strain P620 was mixed with ferulic acid (FA)-decomposing and PHBA-degrading *Pseudomonas* sp. CFA, the mixture was inoculated into cucumber-planted soil supplemented with FA, PHBA, and nitrate. Compared to the uninoculated strain, co-inoculation with strains CFA and P620 improved cucumber growth and increased the activities of antioxidant enzymes in leaves under FA, PHBA, and nitrate. Meanwhile, co-inoculation decreased the concentrations of FA, PHBA, and nitrate in the soil, enhanced the activities of soil enzymes, including sucrase, urease, and catalase, and altered the abundance of rhizospheric bacterial communities. The mixed strains of CFA and P620 improved cucumber growth in soil containing realistic levels of phenolic acids and nitrate. We proposed that co-inoculation with CFA and P620 affects rhizospheric bacterial communities; consequently, the co-inoculation degraded phenolic acids in soil, reduced soil nitrate, activated plant antioxidant enzymes, and induced soil enzymes, ultimately mitigating the combined stresses of phenolic acids and nitrate in greenhouse cucumbers.

## Introduction

In greenhouse production systems, crops such as cucumbers are continuously cultured (Zhou et al., 2021). Therefore, the phenolic acids secreted from these crops can accumulate in greenhouse soils (Zhang et al., 2019). In continuous-cropping soil of cucumber, ferulic acid (FA) and p-hydroxybenzoic acid (PHBA) are the main phenolic acids, while low concentrations of benzoic acid and vanillin are found as well (Ma et al., 2005). The accumulation of these phenolic acids causes oxidative stress in crops (Imatomi et al., 2013), leads to autotoxicity, and hinders continuous cropping (Zhang et al., 2010), thereby inhibiting plant growth (Wu et al., 2018). Nitrate also accumulates in the soil of greenhouses owing to the excess application of fertilizers and low efficiency of nitrogen use, which results in nutrient disorders (Wang et al., 2022) and causes oxidative stress in crops (Han et al., 2024). Moreover, accumulated phenolic acids (Ma et al., 2005) and nitrates (Huang et al., 2016) were found in vegetable fields. Therefore, crops in greenhouses and vegetable fields are subjected to the combined stresses of phenolic acids and nitrate. To the best of our knowledge, no previous study has reported an approach for mitigating combined phenolic acid and nitrate stresses in soil-grown plants.

The application of beneficial soil microorganisms represents a cost-effective and environmentally sustainable approach to mitigating environmental stress in plants (Hanaka et al., 2021). In phenolic acid-accumulated soil, inoculation with phenolic acid-degrading bacteria decreases the concentrations of phenolic acids in the soil, induces plant antioxidant enzymes, activates soil enzymes, and alters the composition and structure of rhizospheric bacterial communities, thus mitigating phenolic acid stress in plants (Wu et al., 2018). Among the phenolic acid-degrading bacteria, *Klebsiella oxytoca* P620 (Wu et al., 2021) and *Pseudomonas* sp. CFA (Zhang Y. et al., 2020) isolated in this lab degrade PHBA to acetyl CoA, and the strain CFA of *Pseudomonas* also decomposes FA to acetyl CoA via vanillin and vanillic acid (Zhang Y. et al., 2020). To remove accumulated nitrate, nitrate-reducing bacteria were inoculated into the soil for electrobioremediation (Choi et al., 2009). In *Klebsiella pneumoniae* M5al, *nasFEDCBA* genes are expressed for nitrate and nitrite assimilation (Lin et al., 1994). In contrast, *narGHI* and *nasAB* genes for nitrate reduction and *nirBD* genes for nitrite reduction have been found in *K. pneumoniae* EGD-HP19-C (Pal et al., 2015). Therefore, the nitrate metabolism pathways are different in *Klebsiella* strains. To date, it has not been reported whether the application of microorganisms alleviates the combined damage caused by phenolic acids and nitrate to soil-grown plants.

In this study, the nitrate reduction genes *narGHI* and nitrite reduction genes *nirBD* were identified in the genome of *K. oxytoca* P620, whereas the *nasAB* genes for nitrate reduction were not detected. Therefore, a novel nitrate-metabolism pathway was analyzed in the strain P620 of *Klebsiella*. Given the high abundance of *Pseudomonas* in soil (Wu et al., 2021), strain P620 was co-inoculated with *Pseudomonas* sp. CFA, capable of decomposing several types of phenolic acids, to degrade the phenolic acids in continuous-cropping soil (Zhang Y. et al., 2020). We hypothesized that when cucumber-planted soil is contaminated with phenolic acids and nitrate, the application of the mixed strains of CFA and P620 might influence the composition and structure of rhizospheric bacterial communities. As a result, the mixed-strain application is expected to decrease phenolic acid and nitrate concentrations in the soil, activate plant antioxidant enzymes, and induce soil enzymes, thus alleviating the combined stresses of phenolic acids and nitrate in cucumber. Therefore, we elucidated a microbe-mediated mechanism for mitigating phenolic acid and nitrate stress. To assess practical applicability, we evaluated the mitigation effects of mixed strains under realistic levels of phenolic acids and nitrates in vegetable fields. Our results highlight the nitrate metabolism pathway of *Klebsiella*, unveil the mechanism by which bacterial inoculation mitigates the combined damage of phenolic acids and nitrate to soil-grown plants, and reveal promising strains of CFA and P620 that assist in plant growth in phenolic acid- and nitrate-contaminated soils in greenhouse and vegetable fields.

## Materials and methods

### Detection of a nitrate metabolism pathway of P620

To investigate the nitrate metabolism pathway of P620 by analyzing nitrate metabolites and gene expression, 1 mL of the strain grown exponentially in Luria–Bertani (LB) medium was inoculated into 100 mL of M-9 N medium, consisting of 5 g L^−1^ glucose, 1 g L^−1^ KH_2_PO_4_, 1 g L^−1^ K_2_HPO_4_, 0.2 g L^−1^ MgSO_4_ (Zhang et al., 2015), and 2 g L^−1^ KNO_3_. Then, the strain was incubated at 37 °C and different shaking speeds (60, 90, 120, 150, or 180 rpm). The concentrations of NO_3_^−^, NO_2_^−^, and NH_4_^+^ in the medium were assayed using thymol (Igarashi, 1976), N-(1-naphthyl)-ethylenediamine (Nicholas and Nason, 1957), and Nessler’s reagent (Jeong et al., 2013), respectively. The transcript levels of *narGHI* and *nirBD* genes in P620 cells cultured at 60 or 180 rpm were estimated with reverse transcription quantitative polymerase chain reaction (Zhang Y. et al., 2020). The primers of these genes are listed in Supplementary Table S1.

To confirm the nitrate metabolism pathway of P620, 1 mL of this strain was added into 100 mL of modified M-9 N medium, in which the nitrogen source was changed to different concentrations of KNO_3_ (1, 3, 5, 7, and 9 g L^−1^), NaNO_3_ (1, 3, 5, 7, and 9 g L^−1^), NaNO_2_ (0.01, 0.03, 0.05, 0.07, and 0.09 g L^−1^), (NH_4_)_2_SO_4_ (0.0001, 0.001, 0.01, 0.1, and 1 g L^−1^), or NH_4_Cl (0.0001, 0.001, 0.01, 0.1, and 1 g L^−1^). When P620 was incubated at 37 °C with shaking at 60 or 180 rpm, the concentrations of NO_3_^−^ (Igarashi, 1976), NO_2_^−^ (Nicholas and Nason, 1957), or NH_4_^+^ (Jeong et al., 2013) were measured.

### Analysis of the abilities of CFA and P620 to degrade benzoic acid and vanillin in medium

Low concentrations of benzoic acid and vanillin have been detected in cucumber continuous-cropping soil (Ma et al., 2005); therefore, the benzoic acid- and vanillin-degrading abilities of CFA and P620 were analyzed. One milliliter of CFA or P620 was inoculated into 100 mL of liquid M9 medium (Turner and Rice, 1975) with benzoic acid (3.68 mg L^−1^) or vanillin (1.55 mg L^−1^) (Ma et al., 2005) as the sole carbon source. The two strains were incubated at 37 °C and 180 rpm. The concentrations of benzoic acid or vanillin were determined in medium using high-performance liquid chromatography (HPLC), according to the procedures of Zhang et al. (2015).

### Determination of the abilities of mixed strains to decompose FA, PHBA, and nitrate in medium

To test whether CFA and P620 could be mixed, non-antagonistic relationships between the two strains were evaluated using a streaking technique (Cruz-López et al., 2021; Sandhu et al., 2025). When cultured until OD_600_ reached 0.4, 30 μL of CFA and P620 were inoculated on LB agar medium at a distance of 1 cm and incubated for 4 d.

To obtain the optimum double-strain inocula under conditions of phenolic acids and nitrate, CFA and P620 were separately incubated in liquid LB medium until OD_600_ reached 1.0, and were mixed in varying ratios (2:1, 1:1, 1:2, 1:3, 1:4, and 1:5). Since FA and PHBA are the main phenolic acids in continuous-cropping soil of cucumber (Ma et al., 2005), the mixtures (1 mL) of CFA and P620 were, respectively, inoculated into 100 mL of M-9NFP medium consisting of 114.4 mg L^−1^ FA, 85.6 mg L^−1^ PHBA (Zhang Y. et al., 2020), 1 g L^−1^ KH_2_PO_4_, 1 g L^−1^ K_2_HPO_4_, 0.2 g L^−1^ MgSO_4_, and 0.2 g L^−1^ KNO_3_. Following incubation of the mixed strains at 37 °C and 180 rpm, the concentrations of FA, PHBA (Zhang Y. et al., 2020), and NO_3_^−^ (Igarashi, 1976) in the medium were measured.

To analyze the nitrate-reducing ability of the mixed strains under FA and PHBA conditions, CFA and P620, grown exponentially in LB medium, were mixed in a ratio of 1:4. Subsequently, 1 mL of the mixture was inoculated in 100 mL of liquid medium containing 114.4 mg L^−1^ FA, 85.6 mg L^−1^ PHBA, 1 g L^−1^ KH_2_PO_4_, 1 g L^−1^ K_2_HPO_4_, 0.2 g L^−1^ MgSO_4_, and different concentrations (0.5, 2, 3.5, and 5 g L^−1^) of KNO_3_. Then, the mixed strains were incubated at 37 °C and 180 rpm. The concentrations of FA, PHBA (Zhang Y. et al., 2020), and NO_3_^−^ (Igarashi, 1976) in the medium were measured.

To evaluate the growth of both strains in combination under KNO₃-containing conditions and to detect the impact of CFA on P620’s ability to metabolize nitrate, CFA was tagged with green fluorescent protein (GFP) by introducing pMP2444 (Chen et al., 2015). Then, 1 mL of CFA, P620, and the strain mixture in a ratio of 1:4 were separately inoculated in 100 mL of liquid medium containing 33.12 mg L^−1^ FA, 9.58 mg L^−1^ PHBA, 3.68 mg L^−1^ benzoic acid, 1.55 mg L^−1^ vanillin (Ma et al., 2005), 1.104 g L^−1^ KNO_3_ (Huang et al., 2016), 1 g L^−1^ KH_2_PO_4_, 1 g L^−1^ K_2_HPO_4_, and 0.2 g L^−1^ MgSO_4_. The growth of P620 and GFP-tagged CFA were counted using the spread plate technique (Chen et al., 2015), and the concentrations of NO_3_^−^ were determined in the medium (Igarashi, 1976).

### Investigation of the mitigation effects of mixed strains on the combined stresses of FA, PHBA, and nitrate in soil-grown cucumber

The effects of nitrate concentration on plant growth were measured under FA and PHBA conditions by analyzing stress-related superoxide radicals (O_2_^−^) (Lin et al., 2011) and malonaldehyde (MDA) (Liu et al., 2010). Each cucumber seedling was planted in a plastic pot filled with 400 g of soil (Zhang et al., 2015). At the two-leaf stage, five groups (8 seedlings per group) of cucumber seedlings were separately watered with the solution consisting of FA (125.56 μg g^−1^ soil), PHBA (94.44 μg g^−1^ soil) (Wu et al., 2021), and different concentrations (1, 2, 3, 4, or 5 mg g^−1^ soil) of KNO_3_. Another group of seedlings was watered with autoclaved water as a control. The six groups of seedlings were then grown for 20 d under conditions of 25 °C and 12 h light (600 μmol m^−2^ s^−1^)/12 h dark (Wu et al., 2021). Cucumber growth indices were determined by measuring plant height, leaf area, and shoot fresh weight (Hou et al., 2018). In addition, the levels of O_2_^−^ (Elstner and Heupel, 1976) and MDA (Xu et al., 2008) were determined in cucumber leaves to detect the stress caused by FA, PHBA, and nitrate.

The effects of inoculum amounts on plant growth were subsequently analyzed under the combined stress of FA, PHBA, and nitrate. Five groups (8 seedlings per group) of cucumber seedlings at the two-leaf stage were separately watered with the solution consisting of FA (125.56 μg g^−1^ soil), PHBA (94.44 μg g^−1^ soil), KNO_3_ (3 mg g^−1^ soil), and different concentrations (0, 3 × 10^5^, 3 × 10^6^, 3 × 10^7^, or 3 × 10^8^ cfu g^−1^ soil) of mixed strains (CFA: P620 = 1:4). Another group of seedlings was watered with autoclaved water and used as the control. When all seedlings were treated for 20 d, plant growth indices were analyzed (Hou et al., 2018), and the levels of O_2_^−^ (Elstner and Heupel, 1976) and MDA (Xu et al., 2008) were measured in the leaves.

CFA (Chen et al., 2015) and P620 (Wu et al., 2021) were tagged with GFP to detect the mitigating effects of the mixed strains on the combined stresses of FA, PHBA, and nitrate in soil-grown cucumbers. Six groups of cucumber seedlings (8 seedlings per group) at the two-leaf stage were treated as follows: (1) control - watered with autoclaved water; (2) CFA+P620 - one group of seedlings was watered with mixed strains (3 × 10^7^ cfu g^−1^ soil) composed of P620 (2.4 × 10^7^ cfu g^−1^ soil) and GFP-tagged CFA (0.6 × 10^7^ cfu g^−1^ soil) and could indicate CFA colonization; another group was watered with mixed strains (3 × 10^7^ cfu g^−1^ soil) consisted of CFA (0.6 × 10^7^ cfu g^−1^ soil) and GFP-tagged P620 (2.4 × 10^7^ cfu g^−1^ soil) and could indicate P620 colonization; (3) FA+PHBA+KNO_3_ - watered with the solution composed of FA (125.56 μg g^−1^ soil), PHBA (94.44 μg g^−1^ soil), and KNO_3_ (3 mg g^−1^ soil); (4) CFA+P620+FA+PHBA+KNO_3_ - one group of seedlings was watered with the solution that consisted of FA (125.56 μg g^−1^ soil), PHBA (94.44 μg g^−1^ soil), KNO_3_ (3 mg g^−1^ soil), P620 (2.4 × 10^7^ cfu g^−1^ soil), and GFP-tagged CFA (0.6 × 10^7^ cfu g^−1^ soil) and could reveal CFA colonization; another group was watered with the solution composed of FA (125.56 μg g^−1^ soil), PHBA (94.44 μg g^−1^ soil), KNO_3_ (3 mg g^−1^ soil), CFA (0.6 × 10^7^ cfu g^−1^ soil), and GFP-tagged P620 (2.4 × 10^7^ cfu g^−1^ soil) and could reveal P620 colonization. Soil moisture was maintained by adding 10 mL of autoclaved water twice daily. Cucumber growth indexes were determined (Hou et al., 2018) after 20 d of cultivation at 25 °C under 12 h light (600 μmol m^−2^ s^−1^)/12 h dark (Wu et al., 2021) conditions. The second leaves and rhizospheric soil of each treatment were collected (Zhang et al., 2015) to analyze the stress mitigation mechanism of the mixed strains of CFA and P620. Three replicates were performed for each treatment.

### Investigation of a mechanism for mitigating the combined stresses of FA, PHBA, and nitrate in soil-grown cucumber

To assess the mitigation effects of mixed strains, colonization of CFA and P620 in rhizospheric soil was evaluated on M9 agar supplemented with PHBA (85.6 mg L^−1^) as the sole carbon source (Wu et al., 2021) by counting GFP-expressing colonies (Chen et al., 2015). Meanwhile, the colonies growing on the M9 medium, excluding GFP-expressing CFA and P620, were considered other phenolic acid-degrading microorganisms.

To analyze phenolic acid degradation and nitrate reduction following application of the mixed strains, the concentrations of phenolic acids and NO_3_^−^ in the rhizospheric soil were determined via HPLC (Chen et al., 2015; Zhang et al., 2015) and phenol-disulfonic acid (Nicholas and Nason, 1957), respectively.

The levels of O_2_^−^ (Elstner and Heupel, 1976) and MDA (Xu et al., 2008) in cucumber leaves were measured to detect stress mitigation in cucumber. The activities of the stressed-related antioxidant enzymes including guaiacol peroxidase (GPX) (Ramiro et al., 2006), catalase (CAT) (Aebi, 1984), glutathione peroxidase (GSH-Px) (Xue et al., 2001), ascorbate peroxidase (APX) (Zhu et al., 2004), dehydroascorbate reductase (DHAR) (Dalton et al., 1993), monodehydroascorbate reductase (MDHAR) (Doulis et al., 1997), and glutathione reductase (GR) (Foyer and Halliwell, 1976) were assayed in cucumber leaves.

Bacterial DNA was extracted from the rhizospheric soil to analyze whether mixed strains could mitigate the combined stresses of FA, PHBA, and nitrate by regulating rhizospheric bacterial communities (Wu et al., 2021). High-throughput sequencing and data processing for 16S rRNA genes of rhizospheric bacterial communities were conducted according to Tao et al. (2017) at Personal Biotechnology Co., Ltd. (Shanghai, China). Rarefaction curves at the 97% similarity level, principal component analysis, and investigation of microbial percentages were performed using the Quantitative Insights Into Microbial Ecology software. Bipartite networks of the top 50 species were constructed using Mothur and Cytoscape software. The relative abundance of functional genes was predicted by mapping reads against the Kyoto Encyclopedia of Genes and Genomes (KEGG) pathway and MetaCyc databases.

Soil microbes affect soil enzyme activities (Schnecker et al., 2015), and the activities of soil enzymes such as sucrase (Schinner and von Mersi, 1990), urease (Tabatabai and Tabatabai and Bremner, 1972), and catalase (Roberge, 1978) were determined in rhizospheric soil to confirm the effects of rhizospheric bacterial communities following mixed-strain application.

### Analysis of the effects of mixed strains on seed germination under the combined stresses of FA, PHBA, and nitrate

To verify the mitigating effects of the mixed strains on the combined stresses of FA, PHBA, and nitrate, cucumber seeds were co-inoculated with CFA and P620. The effects of different concentrations of KNO_3_ on seed germination were examined. Cucumber seeds were soaked into autoclaved water for 12 h and then placed on autoclaved filter paper moistened with different concentrations (4, 6, 8, 10, and 12 g L^−1^) of KNO_3_.

The effects of different concentrations of KNO_3_ were investigated using FA and PHBA. When being soaked with autoclaved water for 12 h, cucumber seeds were put on filter paper, which was moistened with the solution consisting of 134.4 mg L^−1^ FA, 85.6 mg L^−1^ PHBA (Zhang Y. et al., 2020), and different concentrations (4, 6, 8, 10, and 12 g L^−1^) of KNO_3_. As the control, cucumber seeds were placed on a filter paper moistened with autoclaved water.

To detect the mitigating effects of mixed strains on the combined stresses of FA, PHBA, and nitrate in cucumber seeds, cultures of CFA and P620 were mixed in a ratio of 1:4. Two groups of sterilized seeds (60 seeds per group) were added to the strain mixture and shaken at 180 rpm for 12 h. Simultaneously, another two groups of sterilized seeds were soaked in liquid LB medium for 12 h and untreated with strains. Then, one group of strain-treated and one group of strain-untreated seeds were separately placed on filter paper moistened with the solution containing FA (125.56 mg L^−1^), PHBA (94.44 mg L^−1^) (Wu et al., 2021), and KNO_3_ (6 g L^−1^). They were designated “CFA+P620+FA+PHBA+KNO_3_” and “FA+PHBA+KNO_3_” treatments, respectively. The remaining strain-treated and strain-untreated seeds were, respectively, placed on filter paper moistened with autoclaved water. They were designated “CFA+P620 treatment” and “control,” respectively.

For each treatment group, three replicates of 60 seeds each were used. During the treatment of the cucumber seeds, the filter paper was kept wet by adding autoclaved water. After treating cucumber seeds on filter paper for 6 d, their fresh weights, hypocotyl lengths, and root lengths were determined.

### Detection of the effects of mixed strains on soil-grown cucumber under the realistic levels of phenolic acids and nitrate

To assess the mitigation effects of mixed strains under the realistic levels of phenolic acids (Ma et al., 2005) and NO_3_^−^ (Huang et al., 2016) in vegetable fields, six groups of cucumber seedlings (8 seedlings per group) at the two-leaf stage were selected to impose the following treatments: I (control), watered with autoclaved water; II (CFA+P620), one group of seedlings was watered with mixed strains (3 × 10^7^ cfu g^−1^ soil) consisted of P620 (2.4 × 10^7^ cfu g^−1^ soil) and GFP-tagged CFA (0.6 × 10^7^ cfu g^−1^ soil) and could show CFA colonization; another group was watered with mixed strains (3 × 10^7^ cfu g^−1^ soil) composed of CFA (0.6 × 10^7^ cfu g^−1^ soil) and GFP-tagged P620 (2.4 × 10^7^ cfu g^−1^ soil) and could reveal P620 colonization; III (phenolic acids+KNO_3_), watered with the solution composed of KNO_3_ (1.104 mg g^−1^ soil), FA (33.12 μg g^−1^ soil), PHBA (9.58 μg g^−1^ soil), benzoic acid (3.68 μg g^−1^ soil), and vanillin (1.55 μg g^−1^ soil) (Ma et al., 2005) and would result in an NO_3_^−^ concentration of 0.677 mg g^−1^ soil (Huang et al., 2016); IV (CFA+P620+phenolic acids+KNO_3_), one group of seedlings was watered with the solution consisted of FA (33.12 μg g^−1^ soil), PHBA (9.58 μg g^−1^ soil), benzoic acid (3.68 μg g^−1^ soil), vanillin (1.55 μg g^−1^ soil), KNO_3_ (1.104 mg g^−1^ soil), P620 (2.4 × 10^7^ cfu g^−1^ soil), and GFP-tagged CFA (0.6 × 10^7^ cfu g^−1^ soil) and could indicate CFA colonization; another group was watered with the solution composed of FA (33.12 μg g^−1^ soil), PHBA (9.58 μg g^−1^ soil), benzoic acid (3.68 μg g^−1^ soil), vanillin (1.55 μg g^−1^ soil), KNO_3_ (1.104 mg g^−1^ soil), CFA (0.6 × 10^7^ cfu g^−1^ soil), and GFP-tagged P620 (2.4 × 10^7^ cfu g^−1^ soil) and could show P620 colonization. Each treatment was repeated thrice.

When cucumber seedlings were grown for 20 d, growth indices, including plant height, leaf area, and shoot fresh weight, were determined (Hou et al., 2018). The concentrations of NO_3_^−^ (Nicholas and Nason, 1957) and phenolic acids, including FA, PHBA, benzoic acid, and vanillin (Zhang Y. et al., 2020), were measured in the rhizospheric soil. Colonization of CFA and P620 in rhizospheric soil was evaluated on M9 agar supplemented with PHBA (85.6 mg L^−1^) as the sole carbon source (Wu et al., 2021) by counting GFP-expressing colonies (Chen et al., 2015). In parallel, the colonies growing on the M9 medium, except for GFP-expressing CFA and P620, were considered other phenolic acid-degrading microbes.

### Statistical analysis

Data are presented as means ± standard error. One-way analysis of variance and the least significant difference was performed using SPSS 22.0 for Windows (SPSS, Inc., Chicago, IL, USA) to analyze differences among treatments. *p* values of <0.05 were considered significant.

## Results

### A nitrate metabolism pathway of P620

**Figure 1:**
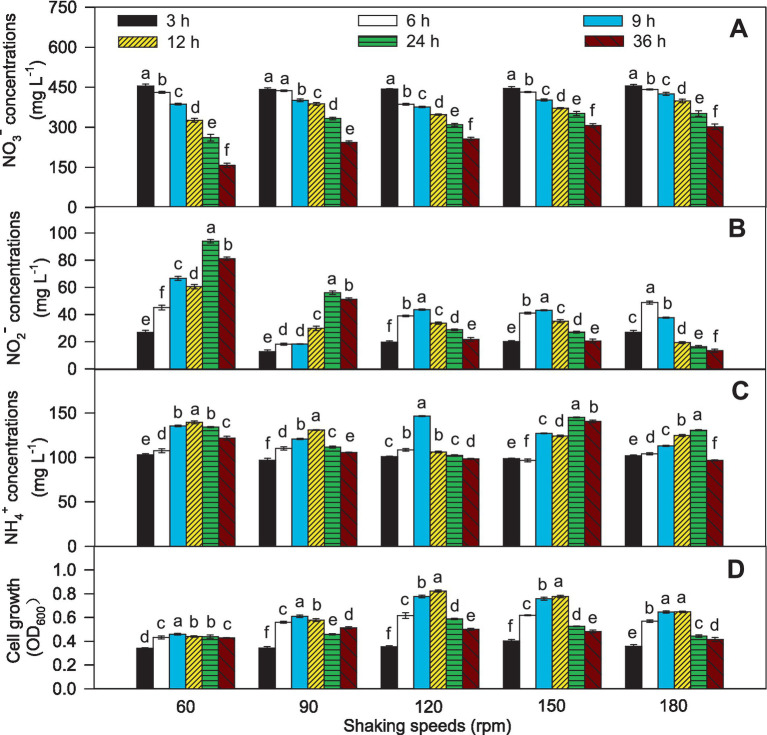
Concentrations of NO_3_^−^ **(A)**, NO_2_^−^ **(B)**, and NH_4_^+^ **(C)** in the medium and growth of cells **(D)** after inoculation with strain P620 into KNO_3_-containing medium. Error bars indicate the standard error of triplicate experiments. At each shaking speed, the means with different letters were significantly different at *p* < 0.05.

**Figure 2:**
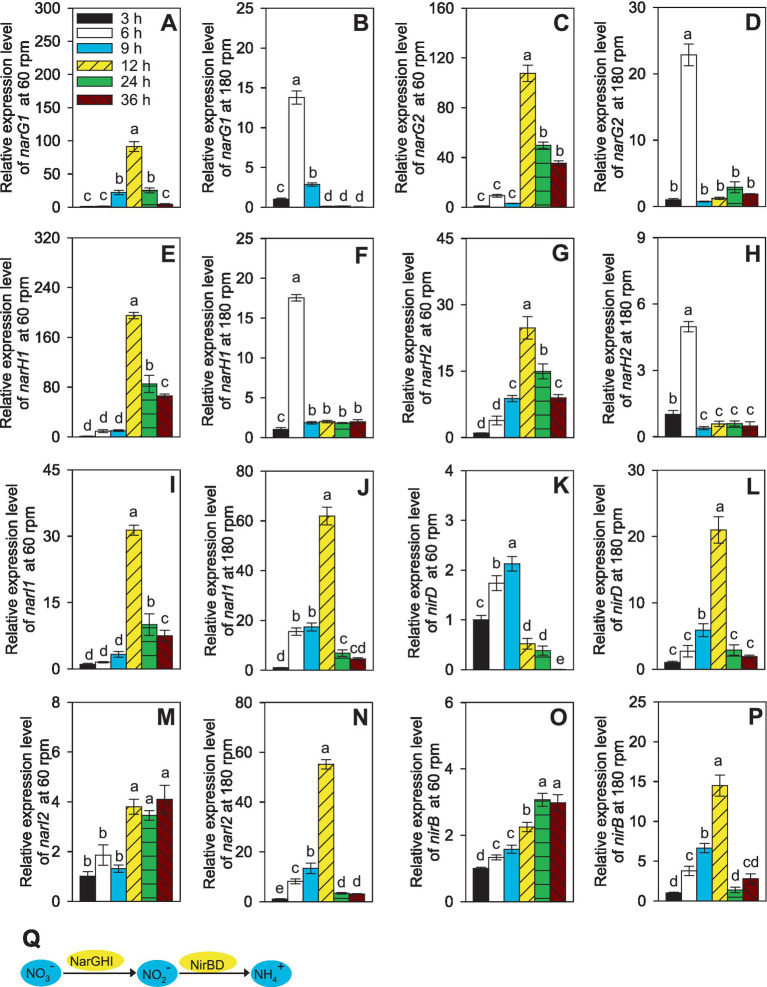
Transcript levels of *narG1* **(A,B)**, *narG2* **(C,D)**, *narH1* **(E,F)**, *narH2* **(G,H)**, *narI1* **(I,J)**, *nirD* **(K,L)**, *narI2* **(M,N)**, *nirB* **(O,P)** in cells after inoculation with the strain P620 into KNO_3_-containing medium at 60 and 180 rpm, and the proposed nitrate metabolism pathway of P620 **(Q)**. Error bars indicate the standard error of triplicate experiments. At each shaking speed, the means with different letters were significantly different at *p* < 0.05.

When P620 was inoculated into KNO_3_-containing medium and incubated at 60–180 rpm, the NO_3_^−^ concentration decreased over time, whereas the NO_2_^−^ and NH_4_^+^ concentrations initially increased and subsequently decreased (Figure 1). At 60 or 180 rpm, the expression levels of *narG1*, *narG2*, *narH1*, *narH2*, *narI1*, and *nirD* in KNO_3_-subjected P620 initially increased and subsequently decreased with increasing incubation time (Figures 2A–L). At 60 rpm, the expression levels of *narI2* and *nirB* in the strain increased during cell growth, while at 180 rpm, they were initially increased and then decreased (Figures 2M–P). We propose that strain P620 reduced NO_3_^−^ to NO_2_^−^ and further to NH_4_^+^ by expressing *narGHI* and *nirBD* (Figure 2Q).

At 60 rpm and different concentrations of KNO_3_ or NaNO_3_, the percentage of reduced NO_3_^−^ by P620 increased with culture time (Supplementary Figures S1A–D). When P620 was exposed to different NaNO_2_ concentrations and incubated at 60 rpm, the percentage of reduced NO_2_^−^ increased during culture (Supplementary Figures S1E,F). After P620 incubation at 60 rpm with different concentrations of (NH_4_)_2_SO_4_ or NH_4_Cl, the percentage of utilized NH_4_^+^ increased with increasing culture time (Supplementary Figures S1G–J). At 180 rpm and different concentrations of KNO_3_, NaNO_3_, NaNO_2_, (NH_4_)_2_SO_4_, and NH_4_Cl, the percentages of reduced NO_3_^−^, NO_2_^−^, and NH_4_^+^ increased during the cultivation of P620 cells (Supplementary Figure S2). These results indicated that P620 can reduce NO_3_^−^ and NO_2_^−^ and would utilize NH_4_^+^.

### Abilities of CFA and P620 to degrade benzoic acid and vanillin in medium

In M9 medium with benzoic acid or vanillin as the sole carbon source, the percentage of benzoic acid or vanillin degraded by the CFA or P620 strain increased with culture time (Supplementary Figure S3). Thus, both CFA and P620 can degrade benzoic acid and vanillin.

### Phenolic acid-degrading and nitrate-reducing abilities of mixed strains in medium

CFA and P620 were not antagonistic (Supplementary Figure S4A), indicating that the two strains could be mixed. The mixed strains of CFA and P620 at a ratio of 1:4 reduced more nitrate at 24 h than those at other ratios (Supplementary Figure S4B) and exhibited rapid degradation of FA (Supplementary Figure S4C) and PHBA (Supplementary Figure S4D). Therefore, a ratio of 1:4 was selected as the optimum mix for CFA and P620.

When different concentrations (0.5–5 g L^−1^) of KNO_3_ were combined with FA and PHBA, the two phenolic acids were degraded well by the mixed strains (Supplementary Figures S4E,F); however, the percentages of the reduced nitrate decreased with increasing initial KNO_3_ concentrations (Supplementary Figure S4G). This suggests that the mixed strains of CFA and P620 gradually reduced high concentrations of nitrate under FA and PHBA.

Both CFA and P620 cells grew gradually in the medium (Supplementary Figure S4H), whereas CFA did not reduce nitrate (Supplementary Figure S4I). After co-inoculation with CFA and P620, the growth of P620 and the percentage of reduced NO_3_^−^ by P620 were enhanced in comparison to inoculation with strain P620 alone, and the growth of CFA did not change at 2–12 h and increased at 18 h when compared with the inoculation with strain CFA alone. Thus, CFA enhanced the ability of P620 to reduce NO_3_^−^, and the two strains were mutualistic and could be used together as mixed inoculants.

### Mitigation of FA, PHBA, and nitrate stresses in soil-grown cucumber by mixed strains

Compared with the control, the levels of plant growth, including plant height (Supplementary Figure S5A), areas of the third leaves (Supplementary Figure S5B), and shoot fresh weights (Supplementary Figure S5C) were significantly decreased under conditions of FA, PHBA, and KNO_3_, whereas the formation rates of O_2_^−^ in the leaves (Supplementary Figure S5D) were considerably increased. Therefore, the combination of KNO_3_ (1–5 mg g^−1^ soil) with FA and PHBA caused stress in cucumber seedlings. With an increase in the initial KNO_3_ concentration, plant heights, areas of the third leaves, and shoot fresh weights decreased under the conditions of FA and PHBA. Correspondingly, the formation rate of O_2_^−^ and the MDA content in the cucumber leaves gradually increased (Supplementary Figure S5). Thus, higher nitrate concentrations induced more severe stress in the cucumbers exposed to FA and PHBA.

When different concentrations of mixed strains were applied to FA-, PHBA-, and KNO_3_- contaminated soils, one concentration (3 × 10^7^ cfu g^−1^ soil) of the mixed strains resulted in higher levels of plant height, larger areas of the third leaf, and lower MDA content in leaves, which was the optimum amount of inoculum (Supplementary Figure S6).

**Figure 3:**
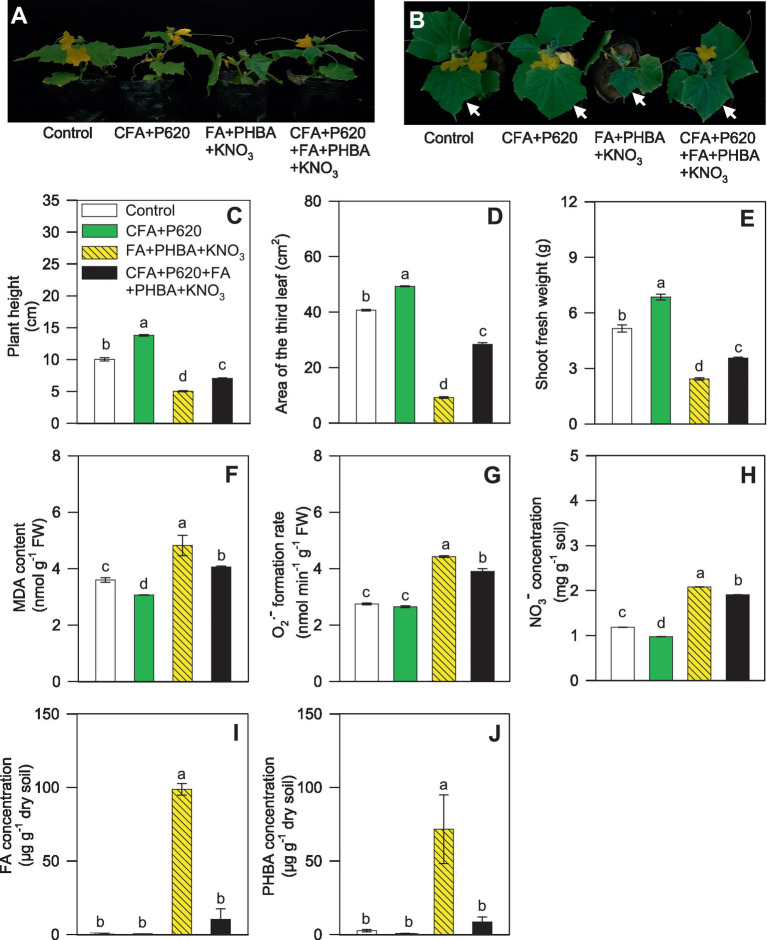
Effects of mixed-strain application on the morphology of cucumber seedlings **(A,B)**, plant height **(C)**, areas of the third leaves **(D)**, shoot fresh weight **(E)**, levels of MDA **(F)** and O_2_^−^ **(G)** in leaves, and concentrations of NO_3_^−^ **(H)**, FA **(I)**, and PHBA **(J)** in soil under the combined stress of FA, PHBA, and KNO_3_. Autoclaved water was used as the control. CFA+P620 was inoculated with mixed strains of CFA and P620. FA+PHBA+KNO_3_, supplemented with FA, PHBA, and KNO_3_. CFA+P620+FA+PHBA+KNO_3_, supplemented with FA, PHBA, and KNO_3_, and inoculated with mixed strains of CFA and P620. The differences between the third leaves of the cucumber seedlings are marked with white arrows. Error bars indicate the standard error of triplicate experiments. Means with different letters are significantly different at *p* < 0.05.

In the FA+PHBA+KNO_3_ treatment, compared to the control, the morphology of cucumber seedlings changed (Figures 3A,B), and the levels of plant height (Figure 3C), areas of the third leaves (Figure 3D), and shoot fresh weights (Figure 3E) were significantly decreased. Meanwhile, the contents of MDA (Figure 3F) and O_2_^−^ (Figure 3G) in the cucumber leaves, and the concentrations of NO_3_^−^ (Figure 3H), FA (Figure 3I), and PHBA (Figure 3J) in the rhizospheric soil were notably higher. In the CFA+P620 treatment, the levels of plant height, areas of the third leaves, and shoot fresh weights were dramatically elevated when compared to the control, whereas the contents of MDA in leaves and the concentrations of NO_3_^−^ in the rhizospheric soil were markedly reduced. The formation rates of O_2_^−^ in cucumber leaves, and the concentrations of FA and PHBA in the rhizospheric soil, remained unchanged between the CFA+P620 and control treatments. Compared with the treatment with FA+PHBA+KNO_3_, treatment with CFA+P620+FA+PHBA+KNO_3_ dramatically increased plant height, third leaf area, and shoot fresh weight. Meanwhile, the contents of MDA and O_2_^−^ in the leaves, and the concentrations of NO_3_^−^, FA, and PHBA in the rhizospheric soil, substantially decreased. Therefore, the application of the mixed CFA and P620 strains improved plant growth, decreased the levels of MDA and O_2_^−^ in cucumbers, and decreased the concentrations of NO_3_^−^, FA, and PHBA in the rhizospheric soil under the combined stresses of FA, PHBA, and nitrate.

### Effects of mixed-strain application on rhizospheric bacterial communities under FA, PHBA, and nitrate

When low-quality, chimeric, and singleton sequences were removed, the high-quality sequences from the treatments of control, CFA+P620, FA+PHBA+KNO_3_, and CFA+P620+FA+PHBA+KNO_3_ were 71,480, 70,432, 66,383, and 65,384, respectively. The operational taxonomic units (OTUs) of the observed species in the four treatments tended to be flat with increasing sequence numbers (Supplementary Figure S7A), indicating that the sequencing depth in each treatment was sufficient.

**Figure 4:**
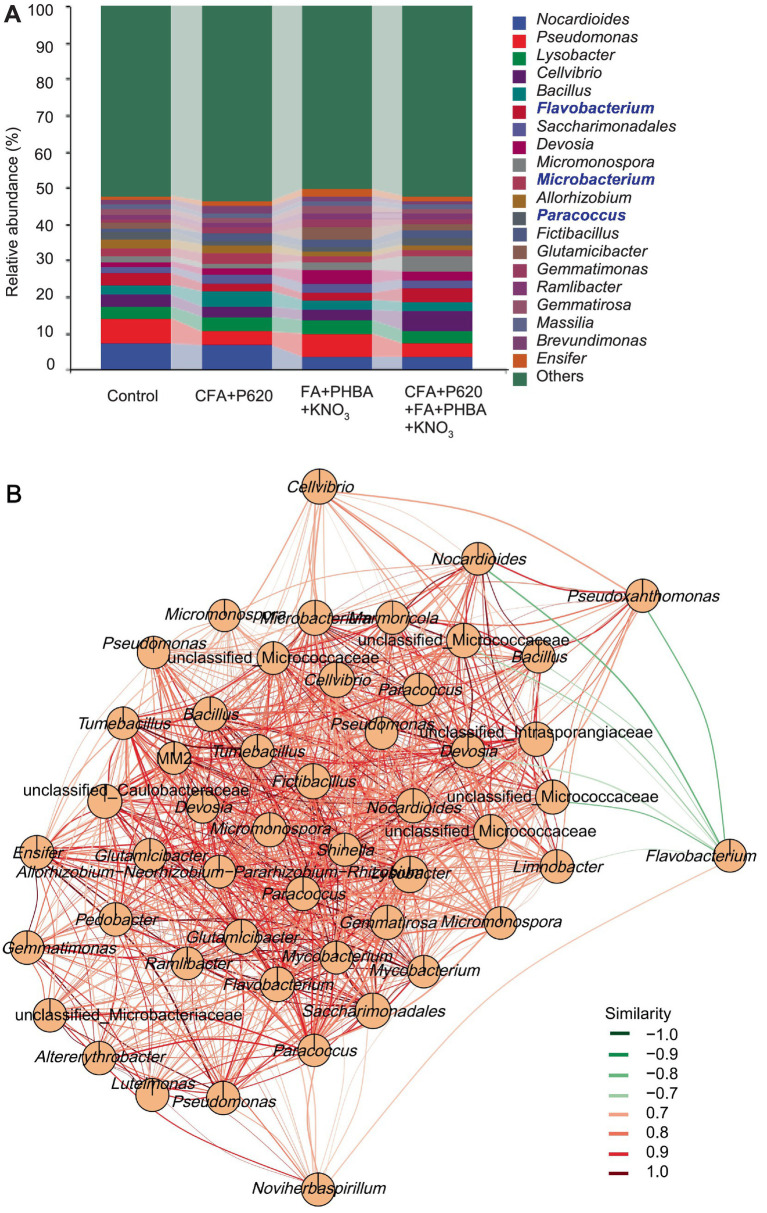
Effects of mixed-strain application on the relative abundances of the top 20 abundant microbial genera under combined stresses of FA, PHBA, and KNO_3_ **(A)** and bipartite network analysis of the microbial genera inferred from the top 50 most abundant OTUs in the CFA+P620+FA+PHBA+KNO_3_ treatment using Mothur and Cytoscape softwares **(B)**. Autoclaved water was used as the control, CFA+P620 was inoculated with mixed strains of CFA and P620. FA+PHBA+KNO_3_, supplemented with FA, PHBA, and KNO_3_. CFA+P620+FA+PHBA+KNO_3_, supplemented with FA, PHBA, and KNO_3_ and inoculated with mixed strains of CFA and P620. The genera that were enriched in the CFA+P620+FA+PHBA+KNO_3_ treatment in comparison to the FA+PHBA+KNO_3_ treatment are marked in blue.

The bacterial communities of the FA+PHBA+KNO_3_ treatment were separated from those of the control or CFA+P620+FA+PHBA+KNO_3_ treatments (Supplementary Figure S7B). The relative abundances of 36 genera (including *Flavobacterium*, *Microbacterium*, *Paracoccus*, *Arthrobacter*, *Rhodoplanes*, and *Chitinophaga*) were considerably higher in the rhizospheric soil of the CFA+P620+FA+PHBA+KNO_3_ treatment than in that of the FA+PHBA+KNO_3_ treatment (Supplementary Table S2; Figure 4A). In addition, the predicted interaction networks for rhizospheric microbes in the CFA+P620+FA+PHBA+KNO_3_ (Figure 4B), control (Supplementary Figure S8), CFA+P620 (Supplementary Figure S9), and FA+PHBA+KNO_3_ treatments (Supplementary Figure S10) differed. Therefore, the abundance, and structure of soil microbes were distinctly affected by the combined stresses of FA, PHBA, and nitrate and the application of mixed strains.

**Figure 5:**
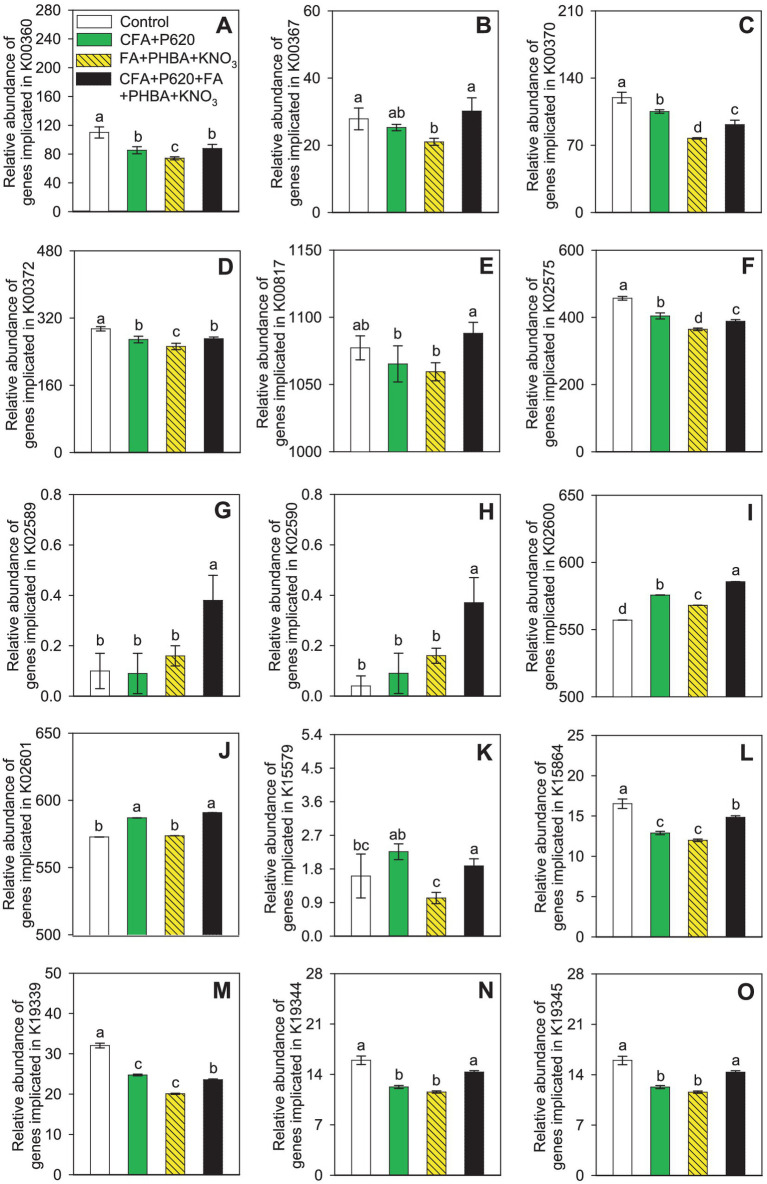
Relative abundances of genes implicated in nitrogen metabolism in rhizospheric bacterial communities. Autoclaved water was used as the control. CFA+P620 was inoculated with mixed strains of CFA and P620. FA+PHBA+KNO_3_, supplemented with FA, PHBA, and KNO_3_. CFA+P620+FA+PHBA+KNO_3_, supplemented with FA, PHBA, and KNO_3_ and inoculated with mixed strains of CFA and P620. Nitrogen metabolism traits were annotated by mapping 16S rRNA amplicon sequencing reads against the KEGG pathway database. K00360, assimilatory nitrate reductase electron transfer subunit; *nasB*. K00367, ferredoxin-nitrate reductase; *narB*. K00370, nitrate reductase/nitrite oxidoreductase alpha subunit; *narG, narZ, nxrA*. K00372, assimilatory nitrate reductase catalytic subunit; *nasA*. K00817, histidinol-phosphate aminotransferase; *hisC*. K02575, nitrate/nitrite transporter; NRT, *narK, nrtP, nasA*. K02589, nitrogen regulatory protein PII 1; *nifHD1, nifI1*. K02590, nitrogen regulatory protein PII 2; *nifHD2, nifI2*. K02600, N utilization substance protein A; *nusA*. K02601, transcriptional antiterminator; *nusG*. K15579, nitrate/nitrite transport system ATP-binding protein; *nrtD, cynD*. K15864, nitrite reductase (NO-forming)/hydroxylamine reductase; *nirS*. K19339, NosR/NirI family transcriptional regulator, nitrous oxide reductase regulator; *nosR*. K19344, cytochrome c55X; *nirC*. K19345, protein NirF; *nirF*. Error bars indicate the standard error of triplicate experiments. Means with different letters are significantly different at *p* < 0.05.

The inoculant survivals of CFA and P620 in rhizospheric soil were separately 5.36 ± 0.10 and 5.86 ± 0.09 log cfu g^−1^ soil in the CFA+P620 treatment and were, respectively, 5.20 ± 0.17 and 5.83 ± 0.13 log cfu g^−1^ soil in the CFA+P620+FA+PHBA+ KNO_3_ treatment. Other phenolic-acid-degrading strains except for CFA and P620 in rhizospheric soil of the CFA+P620 and CFA+P620+FA+PHBA+KNO_3_ treatments were 6.63 ± 0.09 and 6.66 ± 0.13 log cfu g^−1^ soil, separately. Moreover, in the CFA+P620+FA+PHBA+KNO_3_ treatment, in comparison to the FA+PHBA+KNO_3_ treatment, the relative abundances of genes implicated in two traits of vanillate degradation (Supplementary Table S3) and 16 traits of nitrogen metabolism (Figure 5; Supplementary Figure S11A) were dramatically increased, among which a notable increase in the abundance of *nasAB* genes that were not found in strain P620 was observed in rhizospheric bacterial communities. Meanwhile, an elevated abundance of the genus *Flavobacterium*, which was implicated in the nitrate reduction trait, was detected in the rhizosphere soil of the CFA+P620+FA+PHBA+KNO_3_ treatment compared to the FA+PHBA+KNO_3_ treatment (Supplementary Figure S11B). Thus, application of the mixed strains of CFA and P620 into soil not only resulted in the colonization of CFA, P620, and other microbes with the ability to decompose phenolic acids or nitrate but also elevated the abundance of genes related to phenolic acid degradation or nitrate reduction in rhizospheric bacterial communities under the combined stresses of FA, PHBA, and nitrate.

**Figure 6:**
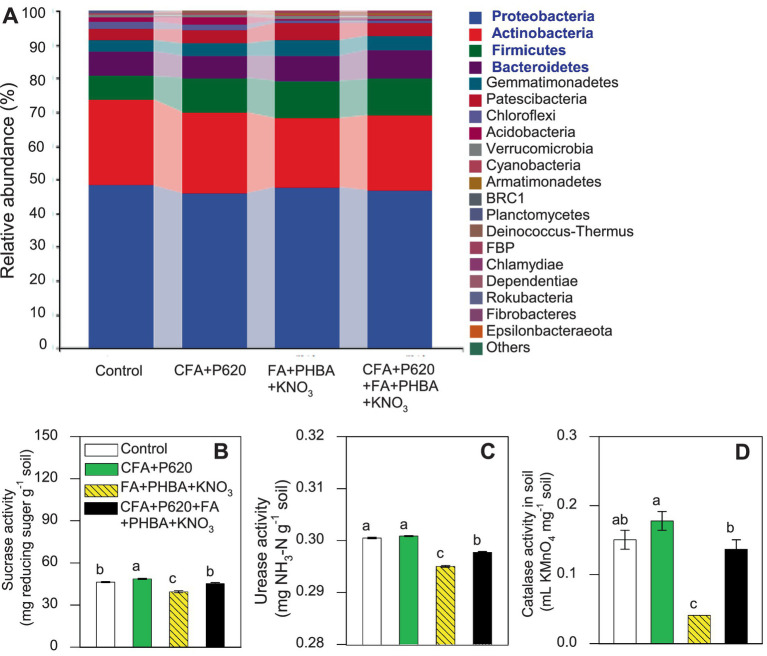
Effects of mixed-strain application on the relative abundances of the top 20 abundant microbial phyla **(A)** and the activities of sucrase **(B)**, urease **(C)**, and catalase **(D)** in rhizospheric soil under combined stresses of FA, PHBA, and KNO_3_. Autoclaved water was used as the control. CFA+P620 was inoculated with mixed strains of CFA and P620. FA+PHBA+KNO_3_, supplemented with FA, PHBA, and KNO_3_. CFA+P620+FA+PHBA+KNO_3_, supplemented with FA, PHBA, and KNO_3_ and inoculated with mixed strains of CFA and P620. Major phyla are indicated in blue. Error bars indicate the standard error of triplicate experiments. Means with different letters are significantly different at *p* < 0.05.

In rhizospheric soil, the most abundant phyla were Proteobacteria, Actinobacteria, Firmicutes, and Bacteroidetes, which accounted for 48.19, 26.85, 7.01, and 6.54% in the control; 48.36, 22.69, 9.70, and 6.28% in the CFA+P620 treatment; 47.88, 21.00, 10.06, and 7.28% in the FA+PHBA+KNO_3_ treatment; and 46.64, 22.93, 10.32, and 8.63% in the CFA+P620+FA+PHBA+KNO_3_ treatment (Figure 6A).

### Effects of mixed-strain application on the activities of soil enzymes under FA, PHBA, and nitrate

The activities of sucrase (Figure 6B), urease (Figure 6C), and catalase (Figure 6D) in the rhizospheric soil were notably decreased under the FA+PHBA+KNO_3_ treatment compared with the control. Compared to the control, the activities of sucrase in the rhizospheric soil were significantly enhanced in the CFA+P620 treatment. The activities of urease and catalase in the rhizospheric soil had no difference between the CFA+P620 treatment and the control. When the CFA+P620+FA+PHBA+KNO_3_ treatment was compared with the FA+PHBA+KNO_3_ treatment, the activities of sucrase, urease, and catalase were significantly increased in the rhizospheric soil. These findings suggested that co-inoculation with CFA and P620 induces soil enzyme activity under the combined stresses of FA, PHBA, and nitrate.

### Effects of mixed-strain application on the activities of plant antioxidant enzymes under FA, PHBA, and nitrate

**Figure 7:**
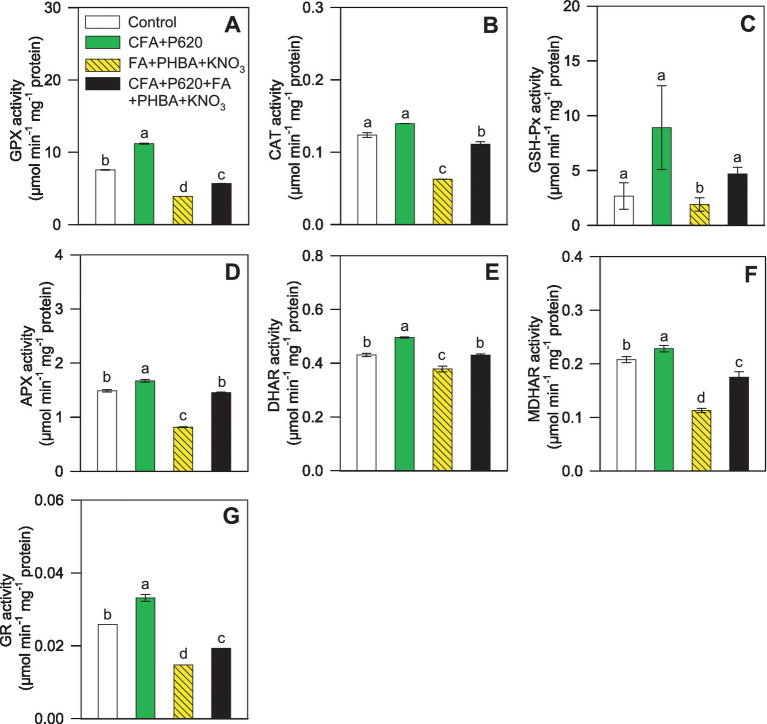
Effects of mixed-strain application on the activities of GPX **(A)**, CAT **(B)**, GSH-Px **(C)**, APX **(D)**, DHAR **(E)**, MDHAR **(F)**, and GR **(G)** in cucumber leaves under combined stress of FA, PHBA, and KNO_3_. Autoclaved water was used as the control. CFA+P620 was inoculated with mixed strains of CFA and P620. FA+PHBA+KNO_3_, supplemented with FA, PHBA, and KNO_3_. CFA+P620+FA+PHBA+KNO_3_, supplemented with FA, PHBA, and KNO_3_, and inoculated with mixed strains of CFA and P620. Error bars indicate the standard error of triplicate experiments. Means with different letters are significantly different at *p* < 0.05.

Compared to the control, the activities of GPX (Figure 7A), CAT (Figure 7B), GSH-Px (Figure 7C), APX (Figure 7D), DHAR (Figure 7E), MDHAR (Figure 7F), and GR (Figure 7G) were significantly lower in cucumber leaves treated with FA+PHBA+KNO_3_. In the CFA+P620 treatment, the activities of GPX, APX, DHAR, MDHAR, and GR were considerably elevated in cucumber leaves compared with those in the control. The activities of CAT and GSH-Px in cucumber leaves did not alter between the CFA+P620 and control treatments. In comparison to the FA+PHBA+KNO_3_ treatment, the activities of GPX, CAT, GSH-Px, APX, DHAR, MDHAR, and GR were substantially increased in the cucumber leaves in the CFA+P620+FA+PHBA+KNO_3_ treatment group. Thus, co-inoculation with CFA and P620 increased the activity of cucumber antioxidant enzymes under the combined stresses of FA, PHBA, and nitrate.

### Mitigation of FA, PHBA, and nitrate stresses in germinating seeds by mixed strains

Regardless of the presence of phenolic acids, the fresh weight, hypocotyl length, and root length of germinating seeds decreased with increasing initial KNO_3_ concentrations (Supplementary Figures S12, S13), indicating that higher concentrations of KNO_3_ caused more severe stress in germinating cucumber seeds.

Fresh weight, hypocotyl length, and root length of germinating seeds were significantly decreased when the FA+PHBA+KNO_3_ treatment was compared to the control but were significantly increased in the CFA+P620+FA+PHBA+KNO_3_ treatment relative to the FA+PHBA+KNO_3_ treatment (Supplementary Figure S14). Thus, the application of mixed strains of CFA and P620 improved the growth of germinating cucumber seeds under the combined stress of FA, PHBA, and nitrate.

### Effects of mixed strains on soil-grown cucumber seedlings under realistic levels of phenolic acids and nitrate

Compared with the control, the phenolic acids+KNO_3_ treatment altered the morphology of cucumber seedlings and significantly decreased the levels of plant heights and areas of the third leaves (Supplementary Figure S15). Meanwhile, the concentrations of NO_3_^−^, FA, PHBA, benzoic acid, and vanillin in the rhizospheric soil were significantly higher. There was no difference in shoot fresh weight between the phenolic acids+KNO_3_ and control treatments. In the CFA+P620 treatment, plant height, areas of the third leaves, and shoot fresh weights were significantly improved when compared to the control, and the concentrations of NO_3_^−^ and vanillin were dramatically decreased in the rhizospheric soil. The concentrations of FA, PHBA, and benzoic acid in the rhizospheric soil did not differ between the CFA+P620 and control treatments. Compared to the phenolic acids+KNO_3_ treatment, the CFA+P620+phenolic acids+KNO_3_ treatment significantly increased plant height, third leaf area, and shoot fresh weights. Meanwhile, the concentrations of NO_3_^−^, FA, PHBA, benzoic acid, and vanillin in the rhizosphere soil decreased significantly. Therefore, the application of mixed strains of CFA and P620 under realistic levels of phenolic acids and nitrate improved plant growth and decreased the concentrations of phenolic acids and NO_3_^−^ in the rhizospheric soil.

The inoculant survivals of CFA and P620 and the abundances of other phenolic-acid-degrading strains in rhizospheric soil were separately 5.66 ± 0.10, 5.49 ± 0.20, and 6.74 ± 0.10 log cfu g^−1^ soil in the CFA+P620 treatment and were, respectively, 5.52 ± 0.07, 5.36 ± 0.10, and 6.95 ± 0.02 log cfu g^−1^ soil in the CFA+P620+phenolic acid+KNO_3_ treatment. Thus, the colonization of CFA, P620, and other phenolic acid-degrading microbes was identified after co-inoculation with CFA and P620 under realistic levels of phenolic acids and nitrate.

## Discussion

### P620 shows a nitrate-reduction pathway of Klebsiella

In this study, strain P620 reduced nitrate levels at both low and high shaking speeds. Similarly, shaking speed had no significant effect on nitrate removal by *Pseudomonas* sp. RWX31 (Zhou et al., 2013). *narGHI* and *nasAB* in *K. pneumoniae* EGD-HP19-C reduce nitrate to nitrite (Pal et al., 2015). However, in *K. oxytoca* P620 examined in this study, *nasAB* genes were not detected, and *narGHI* genes were expressed to reduce nitrate. Therefore, nitrate reduction in strain P620 of *Klebsiella* was distinct. Due to the expression of *narGHI* and *nirBD* in strain P620 of the current study, nitrate was converted to NH_4_^+^ via nitrite. This was supported by the fact that P620 utilized nitrate, nitrite, and NH_4_^+^ with different nitrogen sources in this study. Similarly, narGHI is the major dissimilatory nitrate reductase in *Escherichia coli* that reduces nitrate to NH_4_^+^ (Rothery et al., 2001).

### Application with the mixed strains of CFA and P620 mitigates the combined stresses of phenolic acids and nitrate

Improvement of plant growth and reduced MDA and O_2_^−^ in plants are indicators of stress mitigation (Wu et al., 2021). In this study, co-inoculation with strains CFA and P620 improved cucumber growth and decreased the levels of MDA and O_2_^−^ in leaves, thereby mitigating the combined stress of FA, PHBA, and nitrate in cucumbers. This was consistent with the improved seedling growth after the application of the mixed strains of CFA and P620 under realistic levels of phenolic acids and nitrate in this study, which is supported by the result that co-inoculation with CFA and P620 promoted the growth of germinating cucumber seeds under the combined stress of FA, PHBA, and nitrate. Similarly, the application of CFA mitigates the stress induced by FA and PHBA in cucumbers (Zhang Y. et al., 2020). Inoculation with strain P620 alleviates PHBA stress in seedlings (Wu et al., 2021). The application of rhizobacteria mitigates the stress caused by combined microplastic and heavy metal pollution in sorghum (Zhang et al., 2024).

### Application with the mixed strains of CFA and P620 mitigates the combined stresses of phenolic acids and nitrate by affecting rhizospheric bacterial communities

The application of *Rheinheimera pacifica* NYJ regulates the soil microbiome, thereby alleviating NaHCO_3_ stress in cucumbers (Bai et al., 2025). In this study, the inoculation of FA-, PHBA-, and nitrate-contaminated soil with mixed strains of CFA and P620 changed the structure of rhizospheric microbes. This change was consistent with the improved cucumber growth and decreased levels of MDA and O_2_^−^ in cucumber. Moreover, strains of *Flavobacterium* (Yan et al., 2024), *Microbacterium* (Madhaiyan et al., 2010), *Paracoccus* (Sahoo et al., 2019), *Arthrobacter* (Pishchik et al., 2002), *Rhodoplanes* (Miyamoto et al., 2022), and *Chitinophaga* (Mehmood et al., 2022) have plant growth-promoting properties. In this study, the abundances of these six genera increased after application of the mixed strains of CFA and P620 under conditions of FA, PHBA, and nitrate, which was in line with improved cucumber growth. Thus, co-inoculation with CFA and P620 mitigated the combined stress of phenolic acids and nitrate by affecting the structure and abundance of the rhizosphere bacterial communities. This is supported by a previous report showing that inoculation with *Acinetobacter calcoaceticus* CSY-P13 mitigates the stress caused by FA and PHBA in cucumbers by changing the soil bacterial communities (Wu et al., 2018).

### Application with the mixed strains of CFA and P620 decreases the concentrations of phenolic acids and nitrate in soil by affecting rhizospheric bacterial communities

The CFA strain degrades FA and PHBA (Zhang Y. et al., 2020) and was observed to decompose benzoic acid and vanillin in this study. Another strain, P620, decomposes PHBA (Wu et al., 2021) and was confirmed to degrade benzoic acid, decompose vanillin, and reduce nitrates. After application of the mixed strains of CFA and P620 under realistic levels of phenolic acids and nitrate, decreased concentrations of FA, PHBA, benzoic acid, vanillin, and nitrate were found in the rhizospheric soil, and inoculant survival of CFA and P620 was observed. When the mixed strains of CFA and P620 were applied to FA-, PHBA-, and nitrate-contaminated soil, the concentrations of FA, PHBA, and nitrate in the rhizospheric soil decreased, and the colonization of CFA and P620 was detected. We propose that the inoculant survival of CFA and P620 in rhizospheric bacterial communities contributes to phenolic acid degradation and nitrate reduction in phenolic acid- and nitrate-contaminated soils. In the present study, other phenolic-acid-degrading or nitrate-reducing microbes except for CFA and P620 were observed after co-inoculation with CFA and P620 under conditions of FA, PHBA, and nitrate, which was consistent with the decreased concentrations of FA, PHBA, and nitrate in rhizospheric soil, indicating that microbes other than CFA and P620 in rhizospheric bacterial communities might participate in phenolic acid degradation and nitrate reduction in phenolic acid- and nitrate-contaminated soils. Moreover, when the mixed strains of CFA and P620 were exposed to FA, PHBA, and nitrate, the abundance of genes related to phenolic acid degradation or nitrate reduction increased in soil microbes. This is consistent with the altered rhizospheric bacterial communities and reduced concentrations of FA, PHBA, and nitrate in the soil. Therefore, inoculation with mixed strains of CFA and P620 decreased the concentrations of phenolic acids and nitrates in the soil by affecting the rhizospheric bacterial communities. Similarly, the application of PHBA-degrading P620 resulted in PHBA degradation in soil by changing the rhizospheric bacterial community structure (Wu et al., 2021). Consistently, in the present study, after the mixed strains of CFA and P620 were inoculated into FA-, PHBA-, and nitrate-contaminated soils, the concentrations of FA, PHBA, and nitrate were reduced. These reductions were associated with improved cucumber growth, suggesting that the co-inoculation with CFA and P620 alleviated the combined stress of phenolic acids and nitrate by decreasing the concentrations of FA, PHBA, and nitrate in the soil. Therefore, we proposed that the application of mixed strains of CFA and P620 affects rhizospheric bacterial communities. As a result, the concentrations of phenolic acids and nitrate in the soil decreased, and the combined stress of phenolic acids and nitrate was mitigated in cucumbers. Similarly, *Acinetobacter calcoaceticus* CSY-P13 changes the soil bacterial community, leading to FA and PHBA degradation in the soil, thereby alleviating the stresses of FA and PHBA in cucumbers (Wu et al., 2018).

### Application with the mixed strains of CFA and P620 induces soil enzymes by affecting rhizospheric bacterial communities

In this study, the most abundant phyla in the rhizospheric soil were Proteobacteria, Actinobacteria, Firmicutes, and Bacteroidetes. Proteobacteria and Bacteroidetes are correlated with C mineralization rates (Acostamartínez et al., 2010). Firmicutes are involved in soil N and C cycling (Ferreira et al., 2015). Many bacteria belonging to the phyla Proteobacteria, Actinobacteria, Firmicutes, and Bacteroidetes produce catalase (Biyada et al., 2020; Zhang J. et al., 2020; Kim et al., 2019). When the mixed strains of CFA and P620 were inoculated into FA-, PHBA-, and nitrate-contaminated soil in this study, the abundances of Proteobacteria, Actinobacteria, Firmicutes, and Bacteroidetes were altered, and the activities of C-related sucrase, N-related urease, and catalase in the soil were increased. Thus, co-inoculation with CFA and P620 induced soil enzymes by affecting the rhizospheric bacterial communities. This is supported by a previous report that *Streptomyces canus* GLY-P2 alters rhizosphere bacterial communities and activates soil enzymes (Wu et al., 2019). Moreover, soil enzymes play a role in nutrient supply and promotion of plant growth (Sardans and Peňuelas, 2005). In this study, improved cucumber growth and increased activities of sucrase, urease, and catalase in the soil were observed after the application of mixed strains of CFA and P620 under the combined stresses of FA, PHBA, and nitrate. These findings suggest that mixed strains of CFA and P620 can alleviate the combined stresses of phenolic acids and nitrate by inducing soil enzyme activity. It has been proposed that co-inoculation with CFA and P620 induces soil enzymes by affecting rhizospheric bacterial communities, thereby mitigating the combined stress of phenolic acids and nitrate in cucumber plants. Similarly, the application of *Rheinheimera pacifica* NYJ activated soil enzymes and mitigated NaHCO_3_ stress in cucumbers by regulating the soil microbiome (Bai et al., 2025).

### Application with the mixed strains of CFA and P620 activates plant antioxidant enzymes by affecting rhizospheric bacterial communities

To remove overproduced reactive oxygen species (ROS) such as O_2_^−^ under oxidative stress, plants evolve antioxidant enzymes; thus, the enhanced activities of plant antioxidant enzymes and decreased levels of ROS are determined when oxidative stress in plants is alleviated (Wan et al., 2015). In this study, inoculation with mixed strains of CFA and P620 under the combined stresses of FA, PHBA, and nitrate resulted in increased activities of cucumber antioxidant enzymes, reduced levels of O_2_^−^, and improved cucumber growth. Thus, the mixed strain of CFA and P620 mitigated the combined stress of phenolic acids and nitrate by inducing plant antioxidant enzyme activity. Root-associated microbes stimulate plant growth under salt stress by activating plant antioxidant enzymes (Liu et al., 2021). When the mixed strains of CFA and P620 were applied under the combined stresses of FA, PHBA, and nitrate in this study, the soil microbial structure changed, and the activities of cucumber antioxidant enzymes increased, suggesting that the mixed strains of CFA and P620 activated cucumber antioxidant enzymes by changing rhizospheric bacterial communities. We proposed that the application of the mixed strains of CFA and P620 affected rhizospheric bacterial communities; consequently, cucumber antioxidant enzymes were activated, and the combined stresses of phenolic acids and nitrate were alleviated. This is supported by a previous report that inoculation with *Rheinheimera pacifica* NYJ induces plant antioxidant enzymes by regulating the soil microbiome, thereby mitigating NaHCO_3_ stress in cucumbers (Bai et al., 2025).

## Conclusion

Strain P620 reduced nitrate to NH_4_^+^ via nitrite by expressing *narGHI* and *nirBD* genes. When mixed strains of CFA and P620 were inoculated into cucumber-planted soil supplemented with FA, PHBA, and nitrate, the rhizospheric bacterial community structure and abundance were altered, and the concentrations of FA, PHBA, and nitrate decreased in the soil. The activities of cucumber antioxidant enzymes, such as GPX, CAT, GSH-Px, APX, DHAR, MDHAR, and GR, were elevated, and soil enzymes, including sucrase, urease, and catalase, were activated. As a result, cucumber growth was improved, and the combined stresses of FA, PHBA, and nitrate were mitigated. Under realistic levels of phenolic acids and nitrates in vegetable fields, co-inoculation with CFA and P620 improved cucumber growth and decreased the concentrations of phenolic acids and nitrate in the soil. Therefore, the mixed strains of CFA and P620 showed potential for application in promoting crop growth in phenolic acid- and nitrate-contaminated soils in greenhouses and vegetable fields.

## Data Availability

The raw reads of the 16S rRNA genes were deposited in the NCBI database under the accession number PRJNA1253585.
