# Supplementary material for: Klebsiella oxytoca P620 and Pseudomonas sp. CFA co-inoculation modulates rhizospheric bacterial communities to alleviate the combined stresses of phenolic acids and nitrate in cucumber
Source: Front Microbiol. 2026 Jun 10;17:1834918. doi: 10.3389/fmicb.2026.1834918 (PMC13291130; doi:10.3389/fmicb.2026.1834918)
Supplement: Supplementary file 1 [file Data_Sheet_1.doc]

**Supplementary materials**

**Table S1**

**Primers for reverse transcription quantitative PCR to analyze the transcript levels of genes in the nitrate metabolism pathway of P620**

| Gene name | Primer sequence | Amplicon size (bp) |
| --- | --- | --- |
| *narG1* | Forward primer: 5’-CAAACAGAAGGGTGAAAC-3’  Reverse primer: 5’-GTATCCATCCTCCCAATC-3’ | 76 |
| *narG2* | Forward prime: 5’-CTACGGCTTCAACTACTAC-3’  Reverse primer: 5’-TCATCCAGCCAGTCAATA-3’ | 90 |
| *narH1* | Forward prime: 5’-AAGAGATGTACGGTCAGTTC-3’  Reverse primer: 5’-TTGGGTTAAGGCAGTGTT-3’ | 75 |
| *narH2* | Forward prime: 5’-GGAGAATGAGACAGACCT-3’  Reverse primer: 5’-CCTGCTCAATCACTTTCG-3’ | 77 |
| *narI1* | Forward prime: 5’-TTCCATATCGGCATTCTC-3’  Reverse primer: 5’-CCACGAATAAACCCAATG-3’ | 75 |
| *narI2* | Forward prime: 5’-CACCACTAAAGCGGATAT-3’  Reverse primer 5’-:GGAGAACGGAATAGTCAG-3’ | 79 |
| *nirB* | Forward prime: 5’-TCAGACTCGCTATCATTG-3’  Reverse primer: 5’-ACAGAAGACGGTAATATCG-3’ | 101 |
| *nirD* | Forward prime: 5’-GATAAACAGGTGGCGATT-3’  Reverse primer: 5’-AAAGAACGGGTCGATATTG-3’ | 75 |
| *16S rRNA* (the reference gene) | Forward prime: 5’-CAAGGTTAAAACTCAAATGA-3’  Reverse primer: 5’-TTCTCATCTCTGAAAACTTC-3’ | 124 |

**Table S2**

Relative abundances of 36 genera in rhizospheric soil after application with the mixed strains of CFA and P620 under combined stresses of FA, PHBA, and KNO_3_

| Taxa | Control | CFA+P620 | FA+PHBA+KNO_3_ | CFA+P620+FA+PHBA+KNO_3_ |
| --- | --- | --- | --- | --- |
| *Flavobacterium* | 2.69E-2±2.45E-3 a | 2.12E-2±2.78E-4 b | 1.96E-2±1.06E-3 b | 3.10E-2±5.26E-3 a |
| *Microbacterium* | 2.05E-2±1.13E-3 b | 2.49E-2±1.69E-3 a | 1.61E-2±1.99E-3 c | 2.07E-2±5.72E-4 b |
| *Paracoccus* | 1.85E-2±1.53E-3 ab | 1.39E-2±8.08E-4 c | 1.56E-2±3.27E-3 bc | 2.21E-2±1.17E-3 a |
| *Tumebacillus* | 8.06E-3±7.73E-4 c | 1.08E-2±9.68E-4 b | 8.48E-3±9.06E-4 bc | 1.45E-2±1.93E-3 a |
| *Saccharimonadaceae* | 8.92E-3±1.48E-3 a | 8.56E-3±2.14E-4 ab | 4.57E-3±7.75E-4 c | 6.88E-3±7.63E-4 b |
| *Methylobacillus* | 1.13E-2±9.25E-4 a | 4.72E-3±3.57E-4 b | 2.35E-3±1.23E-4 c | 5.53E-3±7.53E-4 b |
| *Arthrobacter* | 2.09E-3±1.92E-4 c | 1.90E-3±5.45E-4 c | 4.20E-3±4.87E-4 b | 7.55E-3±1.20E-3 a |
| *Sericytochromatia* | 1.78E-3±2.84E-4 c | 2.36E-3±4.14E-4 c | 3.79E-3±5.85E-4 b | 5.32E-3±6.75E-4 a |
| *Dyadobacter* | 3.86E-3±3.12E-4 a | 4.04E-3±4.00E-4 a | 1.52E-3±1.12E-4 c | 2.07E-3±1.67E-4 b |
| OM27_clade | 1.04E-3±2.60E-4 c | 1.50E-3±1.43E-4 bc | 1.97E-3±1.99E-4 b | 3.60E-3±4.07E-4 a |
| *Hydrogenophaga* | 1.36E-3±1.04E-4 b | 1.80E-3±3.35E-4 a | 7.85E-4±1.34E-4 c | 1.91E-3±1.29E-4 a |
| Ellin6067 | 9.78E-4±1.05E-4 b | 9.12E-4±1.50E-4 b | 8.24E-4±8.06E-5 b | 1.25E-3±1.74E-4 a |
| Candidatus_*Nucleicultrix* | 2.09E-5±3.61E-5 c | 1.05E-3±1.87E-4 b | 7.56E-4±2.74E-4 b | 1.90E-3±1.20E-4 a |
| *Sphingopyxis* | 6.80E-4±7.46E-5 b | 9.16E-4±1.12E-4 a | 6.73E-4±6.12E-5 b | 8.90E-4±9.41E-5 a |
| *Pelagibacterium* | 4.70E-5±5.86E-5 c | 0.00±0.00 c | 1.25E-3±7.31E-5 b | 1.81E-3±1.42E-4 a |
| *Actinotalea* | 6.65E-4±9.93E-5 b | 9.68E-4±1.71E-4 a | 4.01E-4±8.24E-5 c | 8.10E-4±9.49E-5 ab |
| *Geminicoccus* | 2.79E-4±9.77E-5 b | 4.19E-4±1.86E-4 b | 5.33E-4±1.72E-4 b | 1.09E-3±1.21E-4 a |
| *Skermanella* | 6.00E-4±4.40E-5 a | 2.85E-4±1.96E-4 b | 2.44E-4±1.59E-4 b | 8.50E-4±1.77E-4 a |
| Subgroup_10 | 1.02E-3±4.51E-4 a | 2.25E-4±7.18E-5 bc | 0.00±0.00 c | 5.20E-4±9.51E-5 b |
| *Arcticibacter* | 2.21E-4±2.00E-4 b | 4.21E-4±1.34E-4 ab | 3.60E-4±1.38E-5 b | 6.40E-4±6.60E-5 a |
| *Caenimonas* | 9.46E-5±5.46E-5 b | 3.36E-5±5.81E-5 b | 5.85E-5±7.87E-5 b | 1.32E-3±1.68E-4 a |
| *Anaeromyxobacter* | 1.71E-4±6.52E-5 b | 4.38E-4±1.28E-4 a | 1.01E-4±1.11E-4 b | 6.10E-4±1.52E-4 a |
| *Panacagrimonas* | 4.38E-4±9.40E-5 a | 5.21E-4±1.03E-4 a | 6.23E-5±6.2E-5 c | 2.40E-4±7.92E-5 b |
| *Rhodoplanes* | 3.48E-4±1.44E-4 a | 1.37E-4±1.75E-4 ab | 4.93E-5±8.55E-5 b | 3.00E-4±8.45E-5 a |
| *Sporocytophaga* | 0.00±0.00 b | 3.59E-4±1.43E-4 a | 1.50E-4±8.70E-5 b | 3.20E-4±4.11E-5 a |
| PB19 | 1.58E-5±2.73E-5 c | 7.68E-5±4.71E-5 bc | 1.59E-4±8.78E-5 b | 4.50E-4±9.58E-5 a |
| *Chitinophaga* | 2.88E-5±3.70E-5 c | 9.89E-5±8.57E-5 bc | 1.32E-4±6.21E-6 b | 3.40E-4±4.25E-5 a |
| *Aquicella* | 6.08E-5±5.27E-5 b | 9.85E-5±9.48E-5 b | 2.76E-5±4.78E-5 b | 3.70E-4±1.98E-5 a |
| *Longimicrobium* | 7.42E-5±9.07E-5 ab | 1.62E-4±2.09E-4 ab | 0.00±0.00 b | 2.80E-4±1.05E-4 a |
| *Ferrovibrio* | 3.76E-5±6.50E-5 b | 1.41E-4±6.22E-5 ab | 9.12E-5±7.98E-5 b | 2.40E-4±4.03E-5 a |
| *Neochlamydia* | 0.00±0.00 b | 1.46E-5±2.52E-5 b | 7.53E-5±7.86E-5 b | 2.80E-4±2.84E-5 a |
| *Lacibacter* | 1.01E-4±6.21E-5 ab | 1.18E-4±7.42E-5 a | 0.00±0.00 b | 1.20E-4±7.63E-5 a |
| *Actinomycetospora* | 0.00±0.00 b | 0.00±0.00 b | 6.29E-5±5.68E-5 b | 2.00E-4±5.55E-5 a |
| MB-A2-108 | 1.67E-5±2.89E-5 ab | 4.85E-5±8.41E-5 ab | 1.05E-5±1.81E-5 b | 1.00E-4±1.67E-5 a |
| *Cytophaga* | 0.00±0.00 b | 0.00±0.00 b | 0.00±0.00 b | 7.70E-5±2.62E-5 a |
| *Chiayiivirga* | 0.00±0.00 b | 0.00±0.00 b | 4.60E-6±7.96E-6 b | 3.60E-5±1.76E-5 a |

Control, watered with autoclaved water. CFA+P620, inoculated with the mixed strains of CFA and P620. FA+PHBA+KNO_3_, supplemented with FA, PHBA, and KNO_3_. CFA+P620+FA+PHBA+KNO_3_, supplemented with FA, PHBA, and KNO_3_ and inoculated with the mixed strains of CFA and P620. Means ± SE, *n* = 3. For each genus, different letters indicate statistically significant differences between treatments at *P* < 0.05.

**Table S3**

Relative abundances of genes implicated in two vanillate-degradation traits of rhizospheric microbial communities

| Functional classifications | Control | CFA+P620 | FA+PHBA+KNO_3_ | CFA+P620+FA+PHBA+KNO_3_ |
| --- | --- | --- | --- | --- |
| Vanillate/3-O-methylgallate O-demethylase (K15066) | 13.19±0.18 a | 12.62±0.53 a | 9.46±0.81 b | 13.32±1.18 a |
| Vanillin and vanillate degradation II (PWY-7098) | 68.33±0.89 ab | 71.11±3.11 a | 65.71±0.69 b | 70.70±1.35 a |

Control, watered with autoclaved water. CFA+P620, inoculated with the mixed strains of CFA and P620. FA+PHBA+KNO_3_, supplemented with FA, PHBA, and KNO_3_. CFA+P620+FA+PHBA+KNO_3_, supplemented with FA, PHBA, and KNO_3_ and inoculated with the mixed strains of CFA and P620. Vanillate degradation traits are annotated by mapping the 16 S rRNA amplicon sequencing reads against the KEGG pathway and MetaCyc databases. Means ± SE, *n* = 3. For each vanillate-degradation trait, different letters indicate statistically significant differences between treatments at *P* < 0.05.

**FIGURE S1**

Percentages of the reduced NO_3_^-^ (**A,C**), decomposed NO_2_^-^ (**E**), or utilized NH_4_^+^ (**G,I**) in medium and growth of cells (**B,D,F,H,J**) after inoculation with the strain P620 under different nitrogen sources and incubation at 60 rpm for 3, 6, 9, 12, 24, and 36 h. Error bars indicate standard errors of triplicate experiments. At each concentration of nitrogen source, means with different letters are significantly different at *P* < 0.05.

**FIGURE S2**

Percentages of the reduced NO_3_^-^ (**A,C**), decomposed NO_2_^-^ (**E**), or utilized NH_4_^+^ (**G,I**) in medium and growth of cells (**B,D,F,H,J**) after inoculation with the strain P620 under different nitrogen sources and incubation at 180 rpm for 3, 6, 9, 12, 24, and 36 h. Error bars indicate standard errors of triplicate experiments. At each concentration of nitrogen source, means with different letters are significantly different at *P* < 0.05.

**FIGURE S3**

Percentages of the degraded benzoic acid (**A,B**) and vanillin (**C,D**) by the strain CFA or P620. Error bars indicate standard errors of triplicate experiments. Means with different letters are significantly different at *P* < 0.05.

**FIGURE S4**

Degradation of phenolic acids and reduction of NO_3_^-^ in medium by mixed strains of CFA and P620. (**A**) Non-antagonistic relationship between the strains CFA and P620. (**B,C,D**) Effects of different strain proportions on the percentages of the degraded FA, decomposed PHBA, and reduced NO_3_^-^. (**E,F,G**) Effects of different KNO_3_ concentrations on the percentages of the degraded FA, decomposed PHBA, and reduced NO_3_^-^. (**H**) Growth of CFA and P620 under phenolic acid- and KNO₃-containing conditions. (**I**) Impact of CFA on P620's ability to metabolize nitrate under conditions of phenolic acids. 2:1, 1:1, 1:2, 1:3, 1:4, and 1:5 represent the proportions of the strains CFA and P620 in inocula. Error bars indicate standard errors of triplicate experiments. At each treatment time, means with different letters are significantly different at *P* < 0.05.

**FIGURE S5**

Effects of KNO_3_ concentrations on the plant heights (**A**), areas of the third leaves (**B**), shoot fresh weights (**C**), and O_2_^.-^ formation rates (**D**) and MDA contents (**E**) in the second leaves under FA and PHBA. Control, watered with autoclaved water. FA+PHBA+KNO_3_, supplemented with FA, PHBA, and different concentrations of KNO_3_. Error bars indicate standard errors of triplicate experiments. Means with different letters are significantly different at *P* < 0.05.

**FIGURE S6**

Effects of innoculum amounts of mixed CFA and P620 on the plant heights (**A**), areas of the third leaves (**B**), shoot fresh weights (**C**), and MDA contents (**D**) and O_2_^.-^ formation rates (**E**) in the second leaves under combined stresses of FA, PHBA, and KNO_3_. Control, watered with autoclaved water. CFA+P620+FA+PHBA+KNO_3_, supplemented with FA, PHBA, and KNO_3_ and inoculated with different concentrations of the mixed strains of CFA and P620. Error bars indicate standard errors of triplicate experiments. Means with different letters are significantly different at *P* < 0.05.

**FIGURE S7**

Rarefaction curves at the 97% similarity level (**A**) and principal component analysis performed on the relative abundances of bacterial genera (**B**). Control, watered with autoclaved water. CFA+P620, inoculated with the mixed strains of CFA and P620. FA+PHBA+KNO_3_, supplemented with FA, PHBA, and KNO_3_. CFA+P620+FA+ PHBA+KNO_3_, supplemented with FA, PHBA, and KNO_3_ and inoculated with the mixed strains of CFA and P620. Bars represent standard errors of three replicates.


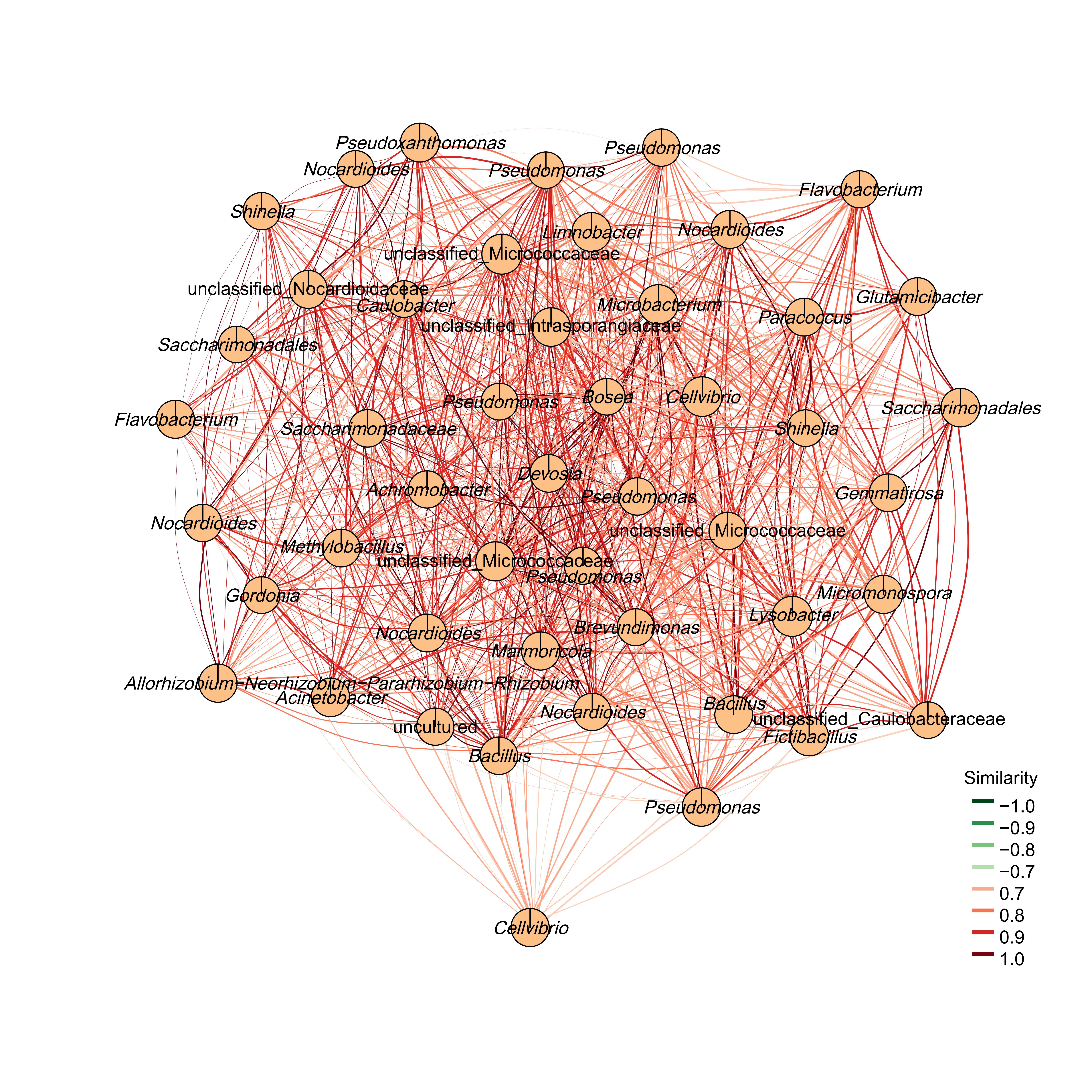


**FIGURE S8**

Bipartite network analysis of genera inferred from the top 50 abundant OTUs in the control treatment by using the Mothur and Cytoscape softwares


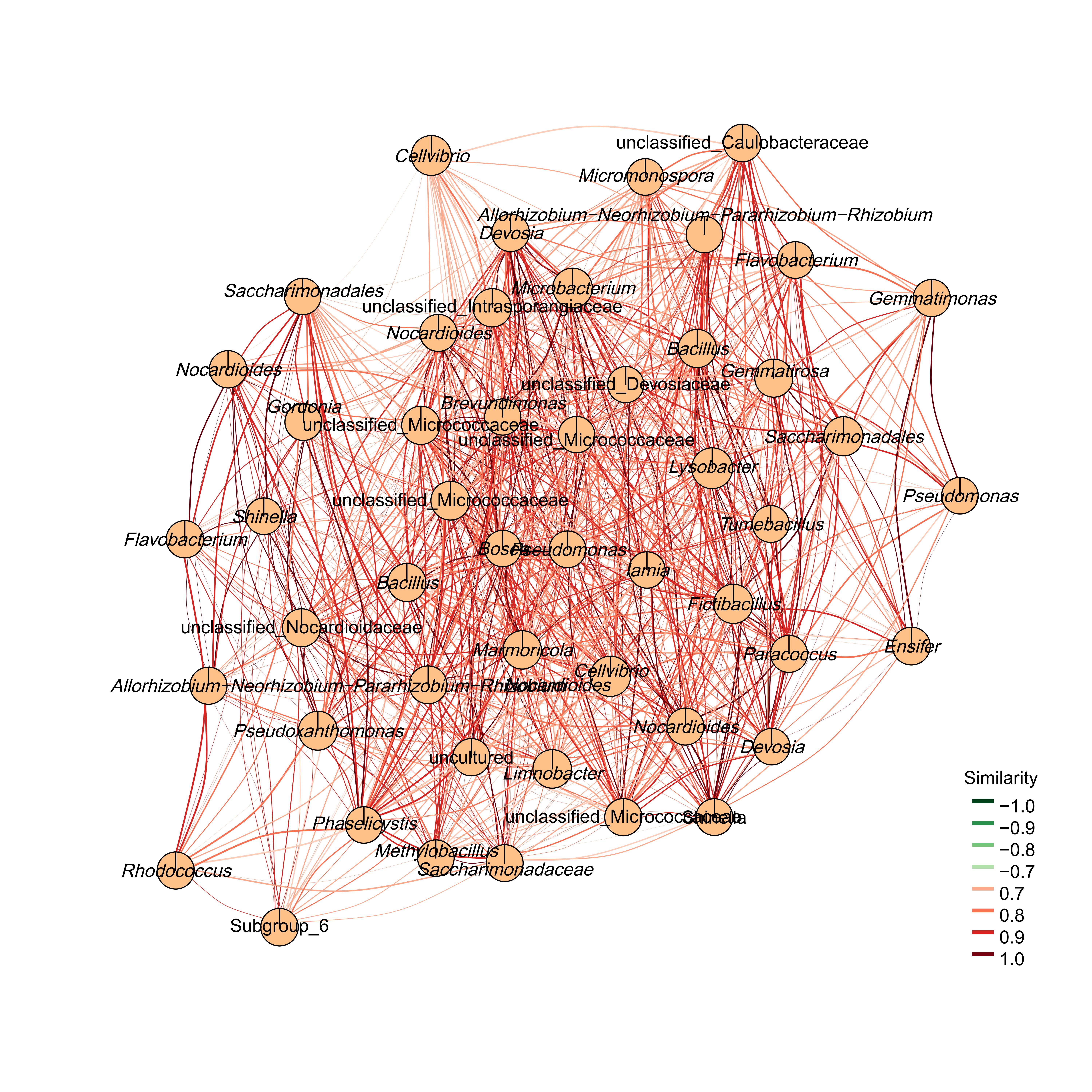


**FIGURE S9**

Bipartite network analysis of genera inferred from the top 50 abundant OTUs in the CFA+P620 treatment by using the Mothur and Cytoscape softwares


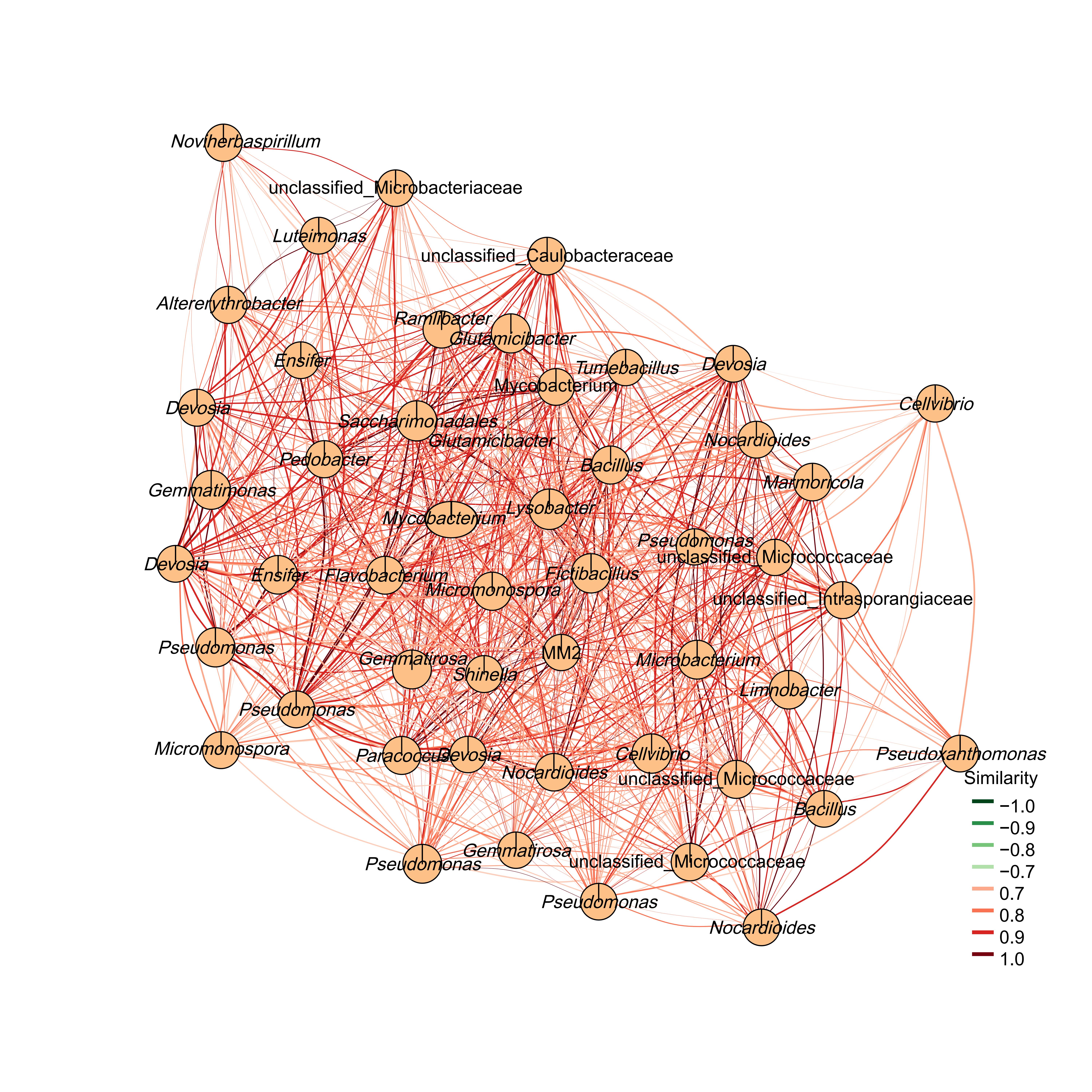


**FIGURE S10**

Bipartite network analysis of genera inferred from the top 50 abundant OTUs in the FA+PHBA+KNO_3_ treatment by using the Mothur and Cytoscape softwares

**FIGURE S11**

Effects of mixed-strain application on the relative abundances of genes (**A**) and the genus *Flavobacterium* (**B**) implicated in PWY490-3 under combined stresses of FA, PHBA, and KNO_3_. Control, watered with autoclaved water. CFA+P620, inoculated with the mixed strains of CFA and P620. FA+PHBA+KNO_3_, supplemented with FA, PHBA, and KNO_3_. CFA+P620+FA+PHBA+KNO_3_, supplemented with FA, PHBA, and KNO_3_ and inoculated with the mixed strains of CFA and P620. PWY490-3 represents nitrate reduction VI (assimilatory) that is annotated by mapping the 16S rRNA amplicon sequencing reads against the MetaCyc database. Error bars indicate standard errors of triplicate experiments. Means with different letters are significantly different at *P* < 0.05.

**FIGURE S12**

Effects of different KNO_3_ concentrations on the seed germination rates (**A**) and the morphology (**B**), fresh weights (**C**), hypocotyl lengths (**D**), and root lengths (**E**) of germinating seeds. Error bars indicate standard errors of triplicate experiments. Means with different letters are significantly different at *P* < 0.05.

**FIGURE S13**

Effects of different KNO_3_ concentrations on the seed germination rates (**A**) and the morphology (**B**), fresh weights (**C**), hypocotyl lengths (**D**), and root lengths (**E**) of germinating seeds under FA and PHBA. Control, exposed to autoclaved water. FA+PHBA+KNO_3_, exposed to FA, PHBA, and different concentrations of KNO_3_. Error bars indicate standard errors of triplicate experiments. Means with different letters are significantly different at *P* < 0.05.

**FIGURE S14**

Effects of mixed-strain application on the seed germination rates (**A**) and the morphology (**B**), fresh weights (**C**), hypocotyl lengths (**D**), and root lengths **E**) of germinating seeds under combined stresses of FA, PHBA, and KNO_3_. Control, exposed to autoclaved water. CFA+P620, treated with the mixed strains of CFA and P620. FA+PHBA+KNO_3_, exposed to FA, PHBA, and KNO_3_. CFA+P620+FA+ PHBA+KNO_3_, treated with the mixed strains of CFA and P620 and exposed to FA, PHBA, and KNO_3_. Error bars indicate standard errors of triplicate experiments. Means with different letters are significantly different at *P* < 0.05.

**FIGURE S15**

Effects of mixed-strain application on the morphology of cucumber seedlings (**A** and **B**), plant heights (**C**), areas of the third leaves (**D**), shoot fresh weights (**E**), and concentrations of NO_3_^-^ (**F**), FA (**G**), PHBA (**H**), benzoic acid (**I**), and vanillin in soil (**J**) under realistic levels of phenolic acids and nitrate. Control, watered with autoclaved water. CFA+P620, inoculated with the mixed strains of CFA and P620. Phenoli cacids+KNO_3_, supplemented with FA, PHBA, benzoic acid, vanillin, and KNO_3_. CFA+P620+phenolic acids+KNO_3_, supplemented with FA, PHBA, benzoic acid, vanillin, and KNO_3_ and inoculated with the mixed strains of CFA and P620. Differences among the third leaves of cucumber seedlings are marked with white arrows. Error bars indicate standard errors of triplicate experiments. Means with different letters are significantly different at *P* < 0.05.
